# Commentary: The SON2A2 score: a novel grading scale for predicting hemorrhage and outcomes after thrombolysis

**DOI:** 10.3389/fneur.2024.1414432

**Published:** 2024-06-17

**Authors:** Andrea Loggini

**Affiliations:** ^1^Brain and Spine Institute, Southern Illinois Healthcare, Carbondale, IL, United States; ^2^Southern Illinois University School of Medicine, Carbondale, IL, United States

**Keywords:** TPA, acute ischemic stroke, symptomatic intracranial hemorrhage, stroke outcome, grading scale

## Introduction

I read with great interest the article titled “The SON2A2 score: a novel grading scale for predicting hemorrhage and outcomes after thrombolysis.” (1). In this original study, the authors developed a grading scale to stratify the risk of symptomatic intracranial hemorrhage and outcome in patients who are candidates to receive thrombolytic therapy for suspected acute ischemic stroke. The SON2A2 score is an 8-point score that takes into account smoking, onset of symptoms, needle time, NIHSS score, neutrophil percentage, age, and ASPECTS (1). The development of an accurate and reliable grading scale that predicts which patients are at higher risk of developing complications from thrombolytics and having poor outcomes is greatly beneficial in clinical practice as an aiding tool in the discussion with patients and family regarding risks and benefits of thrombolytics, as well as triaging patients to specific levels of care after receiving thrombolytic therapy based on their risk of complications. Given the ease of use of the scale and the good accuracy of the model in the original study, I tried to externally validate the SON2A2 score in my patient population. In this attempt, by reviewing in-depth the methods and results of the study, several questions arose, and I hope to receive clarification from the authors.

## ASPECTS

The Alberta Stroke Program Early CT Score (ASPECTS) is a 10-point quantitative topographic CT scan score originally designed to stratify the risk of death based on the initial head CT in patients eligible to receive thrombolytic therapy for ischemic stroke (2). One point is subtracted from a total of 10 points based on the radiographic appearance of certain areas of the territory of the middle cerebral artery on the head CT. The highest score is 10, while the poorest score is 0. A sharp increase in fatal outcomes was observed in the original paper for ASPECTS of 7 or less. ASPECTS, using the same grading scale from 10 to 0, has also been used as an inclusion criterion in trials regarding the use of mechanical thrombectomy within the 6-h timeframe (3–5). In the original study of Ren et al., ASPECTS represents one of the six variables that compose the SON2A2 score (1). In the results of the study, the median (IQR) ASPECTS in the derivation cohort are 13 (12, 14) and 12 (10, 13) for the non-sICH and sICH groups. In the validation cohort, the median (IQR) ASPECTS are 13 (13, 14) and 11 (10, 12) for the non-sICH and sICH groups (1). The cutoff of ASPECTS in the SON2A2 grading scale to predict a poor outcome is 11 (1). These numbers are all outside the possible range of ASPECTS scores, which again ranges between 10 and 0. Therefore, it is impossible to reproduce the SON2A2 score as currently presented.

## Rate of symptomatic intracranial hemorrhage

The rate of symptomatic intracranial hemorrhage (sICH) following thrombolytic therapy within 3 h of the last known well was reported to be ~6% in the NINDS trial (6). In the ECASS 3 trial, which evaluated the use of thrombolytic therapy between 180 and 270 min from the last known well, the rate of sICH in the rTPA group was 2.4% (7). Data regarding the development of sICH after thrombolytic therapy for acute ischemic stroke are comparable in patients treated with tenecteplase compared to alteplase (8). However, in the study of Ren et al., the rate of sICH in the study was reported as 12.2% (68/556) in the derivation cohort and 8.7% (14/160) in the validation cohort (1). It is important to note that 167 patients were excluded from their analysis due to incomplete data, stroke in the posterior fossa, incomplete dose of r-tPA, or severe concomitant diseases. Even assuming that none of the excluded patients developed sICH, the rate of sICH in their cohort is calculated as 9.3% (82/883). Additionally, the methods do not specify the classification used to define sICH. Altogether, the rate of sICH in their cohort appears to be discrepant and out of proportion with what the available literature suggests for stroke patients treated with thrombolytic therapy within 4.5 h from the last known well.

## Last known well to needle time

Finally, thrombolytic therapy is the standard of care for suspected acute ischemic stroke within 4.5 h of symptom onset recognition (9). In the study of Ren et al., the methods section states that one of the inclusion criteria was “R-tPA was administered at the standard dose within 4.5 h after the onset of symptoms.” (1). However, in the validation cohort, the onset of symptoms to treatment, expressed as mean ± standard deviation, in the sICH group was 3.31 ± 1.35 h. The upper bound of this range, translated into minutes, is equivalent to 279 min, which exceeds the timeframe stated in the methods section. In other words, it appears that there were patients included in the study who did not meet the specified inclusion criteria. This may question the validity of the results.

## Discussion

In conclusion, I applaud the effort demonstrated by the authors in developing a predicting grading score for complications following thrombolytic therapy and assessing outcomes in stroke patients, a much-needed advancement in clinical practice. However, the study exhibits discrepancies and limitations that necessitate careful consideration when interpreting its results and applying them in a real-world setting. Further studies are essential to refine the score and enhance its applicability to diverse populations.

## Author contributions

AL: Writing – original draft, Writing – review & editing.
